# Cardiovascular Biomarkers: Current Status and Future Directions

**DOI:** 10.3390/cells12222647

**Published:** 2023-11-17

**Authors:** Mark G. Filipovic, Markus M. Luedi

**Affiliations:** Department of Anesthesiology and Pain Medicine, Inselspital, Bern University Hospital, University of Bern, 3010 Bern, Switzerland; mark.filipovic@insel.ch

Cardiovascular disease (CVD) remains a global health concern of paramount significance, claiming millions of lives each year. While advancements in all fields of cardiovascular science, including but not limited to imaging and interventional techniques, as well as lifestyle modifications and medication for primary and secondary prevention, have bolstered the arsenal for the fight against CVD, the importance of individualized early detection, precise diagnosis and prognosis, and effective treatment is increasingly recognized. Biomarkers play a crucial role in the diagnosis, prognosis, and management of various diseases. In the past decade, tremendous strides have been made in the identification and utilization of cardiovascular biomarkers [1]. Traditional risk markers such as cholesterol [2] have paved the way for more intricate indicators, such as lipoprotein (a) [3] and troponin levels, B-type natriuretic peptides (BNPs [4]), and C-reactive protein (CRP). Some of these biomarkers have been instrumental in diagnosing conditions such as myocardial infarction or heart failure, and even in assessing prognosis, including the risk of future cardiac events [2,3].

However, there are several shortcomings and challenges associated with the current biomarkers, and there are important clinical questions for which biomarkers are still lacking.

The shortcomings of current biomarkers include a lack of specificity, meaning that they can be elevated in multiple diseases, making it challenging to pinpoint the exact cause of a patient’s symptoms. For example, elevated CRP can indicate inflammation but does not specify the source or cause of inflammation [5]. In addition, some biomarkers may not be sensitive enough to detect diseases in their early stages, resulting in delayed diagnosis and treatment, which is especially critical for CVD, where early intervention is often crucial [6]. Further, biomarker levels can vary significantly among individuals, making it difficult to establish universal cutoff values for both diagnosis and risk assessment. Genetic and environmental factors can contribute to this variability [4].

Identifying individuals at high risk of developing CVD is a complex challenge [4]. More reliable biomarkers are needed to better predict an individual’s risk, and tailoring preventive strategies to detect CVDs in their early stages is crucial for timely intervention. Further, CVDs are a heterogeneous group of conditions with complex underlying mechanisms, challenging the identification of universal biomarkers that can cover all aspects of cardiovascular health.

Biomarker research often involves the use of patient data and biological samples. Ethical and privacy concerns can limit the accessibility of such data, hindering the development of robust biomarkers. Biomarker discovery also requires extensive validation and confirmation in diverse patient populations where individual biology may by influenced by a combination of genetic, lifestyle, and environmental factors. Reliable biomarkers need to account for this complexity to be effective.

While we have come far, the journey towards an optimally healthy cardiovascular system continues. In this Special Issue, we explore new horizons to refine our understanding of cardiovascular biomarkers and their potential in the era of personalized medicine, e.g., in Fabry or Marfan disease. Tailoring treatment plans based on an individual’s genetic makeup and unique biomarker profile promises more effective and targeted interventions, e.g., in pro-inflammatory conditions. In addition, machine learning algorithms are being harnessed to analyze vast datasets and identify subtle patterns in biomarker fluctuations. This holds the potential to predict CVD risk with unprecedented accuracy.

Ongoing research is unveiling novel biomarkers that could revolutionize CVD diagnosis and management. These include microRNAs, genetic variants, and metabolomic markers, among others. Cardiovascular biomarkers have already transformed the landscape of CVD diagnosis and management. As we venture into the future, the potential for these biomarkers to save lives and improve the quality of life for countless individuals is boundless. With ongoing research, technologic advancements, and a commitment to prevention, the journey towards healthier hearts and vessels is both exciting and promising. As we continue to explore these avenues, let us remain steadfast in our pursuit of a world where CVD is a relic of the past and cardiovascular health thrives.

This Special Issue aims to provide insights into the current status and future directions of cardiovascular biomarker research.
